# Bioinformatics Analysis of SNPs in IL-6 Gene Promoter of Jinghai Yellow Chickens

**DOI:** 10.3390/genes9090446

**Published:** 2018-09-06

**Authors:** Shijie Xin, Xiaohui Wang, Guojun Dai, Jingjing Zhang, Tingting An, Wenbin Zou, Genxi Zhang, Kaizhou Xie, Jinyu Wang

**Affiliations:** 1 College of Animal Science and Technology, Yangzhou University, Yangzhou 225009, China; shijiexin123@163.com (S.X.); wxh9409161412@126.com (X.W.); zhangjingjing92091@163.com (J.Z.); 18852728062@163.com (T.A.); wenbinzou1216@163.com (W.Z.); zgx1588@126.com (G.Z.); yzxkz168@163.com (K.X.); jywang@yzu.edu.cn (J.W.); 2 Key Lab for Animal Genetics, Breeding, Reproduction and Molecular Design of Jiangsu Province, Yangzhou 225009, China; 3 Institutes of Agricultural Science and Technology Development, Yangzhou University, Yangzhou 225009, China

**Keywords:** Jinghai yellow chicken, *IL-6* gene SNPs, transcription factor, CpG island, bioinformatics

## Abstract

The proinflammatory cytokine, interleukin-6 (IL-6), plays a critical role in many chronic inflammatory diseases, particularly inflammatory bowel disease. To investigate the regulation of *IL-6* gene expression at the molecular level, genomic DNA sequencing of Jinghai yellow chickens (*Gallus gallus*) was performed to detect single-nucleotide polymorphisms (SNPs) in the region −2200 base pairs (bp) upstream to 500 bp downstream of *IL-6*. Transcription factor binding sites and CpG islands in the *IL-6* promoter region were predicted using bioinformatics software. Twenty-eight SNP sites were identified in *IL-6*. Four of these 28 SNPs, three [−357 (G > A), −447 (C > G), and −663 (A > G)] in the 5′ regulatory region and one in the 3′ non-coding region [3177 (C > T)] are not labelled in GenBank. Bioinformatics analysis revealed 11 SNPs within the promoter region that altered putative transcription factor binding sites. Furthermore, the C-939G mutation in the promoter region may change the number of CpG islands, and SNPs in the 5′ regulatory region may influence *IL-6* gene expression by altering transcription factor binding or CpG methylation status. Genetic diversity analysis revealed that the newly discovered A-663G site significantly deviated from Hardy-Weinberg equilibrium. These results provide a basis for further exploration of the promoter function of the *IL-6* gene and the relationships of these SNPs to intestinal inflammation resistance in chickens.

## 1. Introduction

Interleukin-6 (IL-6), also known as interferon β-2, stem cell-stimulating factor, or B cell differentiation factor, is a multifunctional cytokine that plays an important role in regulating the immune response, acute-phase response, and haematopoiesis in chickens [1]. Amrani et al. [2] first discovered that chicken IL-6 influences hepatocyte-stimulating factor (HSF) in the acute-phase response. Schneider et al. [3] successfully cloned the complete reading frame cDNA for chicken *IL-6*. Horiuchi et al. [4] reported that the chicken leukaemia inhibitory factor belongs to the IL-6 family. By injecting DNA plasmids carrying the very virulent infectious bursal disease virus (vvIBDV) *SH95* poly-protein (VP2-4-3) gene and DNA plasmids carrying chicken *IL-6* (*ChIL-6*), Sun et al. [5] determined that injection with ChIL-6 plasmids resulted in a significantly increased protective effect in chickens after infection with highly virulent strains. *IL-6* plays a vital role in the chronic inflammation and pathogenesis of autoimmune diseases, especially inflammatory bowel disease. Neurath and Finotto [6] reported that *IL-6* plays a key role in the regulation of T lymphocyte differentiation and activation by inducing the Jak/STAT-3 and Ras/Erk/C/EBP pathways. Previous studies have demonstrated that *IL-6* exhibits significant potential with respect to the replacement of antibiotics for chickens, and the functional and clinical applications of *IL-6* genes as immunostimulants and immune adjuvants are being actively explored [7,8,9,10]. Swaggerty et al. [11] developed a novel selection method based on the identification and selection of chickens with an inherently high and low phenotype of proinflammatory mediators of *IL-6*; the results from this study showed that in addition to an enhanced resistance against *Salmonella Enteritidis*, high line chickens were also more resistant to the pathology associated with coccidial infections compared to the low line birds. Using an experimental protocol involving *Eimeria tenella* infection, individual resistant and susceptible Jinghai yellow chickens were selected, and caecum transcriptome sequencing of the resistant and susceptible chickens revealed that the differentially expressed genes concentrated in cytokine-cytokine receptor pathway interactions included *IL-6*, *IL-8*, *IL-12β, IL-15, IL-17*, and *TGFB2* [12]. Liu et al. [13] reported that *IL-6* expression patterns in ducks (*Anas platyrhynchos*) with Newcastle disease virus (NDV) were different from those in chickens (*Gallus gallus*); the expression levels of *IL*-6 in the peripheral blood lymphocytes of chickens at 12, 24, and 48 h were higher than those of ducks by 0.157, 4.084, and 0.052-fold, respectively. Therefore, studying the regulation of chicken *IL*-6 gene expression is of great significance.

In the present study, DNA sequencing technology was applied to detect single-nucleotide polymorphisms (SNPs) of the *IL-6* gene in the Jinghai yellow chicken model. Bioinformatics analyses were used to predict whether gene mutations, especially in the 5′ promoter region, would alter transcription factor binding sites and CpG islands. The objective of this study was to provide a basis for validating the influence of SNPs on *IL-6* gene expression and the association between SNPs and coccidiosis resistance in Jinghai yellow chickens.

## 2. Materials and Methods

### 2.1. Experimental Animals

The animals used in this study were obtained from the Jinghai Yellow Chicken Breeding Station in Nantong City, Jiangsu Province, China. The experimental chickens were housed in a sterile animal room and fed an antibiotic-free diet. A total of 220 (110♂ + 110♀) 38-day-old healthy and physiologically similar Jinghai yellow chickens were randomly selected and were used for *IL-6* gene SNP detection and bioinformatics analysis. All animal protocols were approved by the Animal Welfare Committee of Yangzhou University (permit number: SYXK (Su) IACUC 2012-0029), and all efforts were made to minimize the suffering of the chickens.

### 2.2. Genomic DNA Extraction

A 1 mL blood sample was collected from the wing vein of each bird using a vacutainer containing acid citrate dextrose as an anticoagulant. Genomic DNA was extracted and purified using the conventional phenol-chloroform extraction method. An Eppendorf BioPhotometer (Eppendorf Scientific, Hamburg, Germany) was employed to assess the DNA concentration and quality based on UV light absorbance at 260 and 280 nm.

### 2.3. Primer Design and Polymerase Chain Reaction Conditions

Based on the GenBank (https://www.ncbi.nlm.nih.gov/genbank/) chicken genomic DNA sequence (Gene ID: 395337), nine pairs of primers were designed using Primer Premier 5 software (PREMIER Biosoft, Palo Alto, CA, USA). The amplified fragments ranged in size from 565 to 803 base pairs (bp) and were located between −2200 bp upstream and 500 bp downstream of the *IL-6* gene. The primers were synthesized by Sangon Biotech Co. (Shanghai, China), and the primer information is presented in Table 1.

Polymerase chain reaction (PCR) was performed in a total volume of 20 μL, with 10 μL of Taq PCR Master Mix reagent (Takara Bio Inc., Otsu, Japan), 1 μL of template DNA (100 ng/μL), 1 μL each of upstream and downstream primer (10 μmol/L), and 7 μL of ddH_2_O (Takara Bio Inc.). PCR amplification was performed as follows: Preliminary denaturation at 94 °C for 5 min; 35 cycles of denaturation for 30 s at 94 °C, annealing for 30 s at the optimum primer annealing temperature, and elongation for 35 s at 72 °C; and a final extension at 72 °C for 7 min. The samples were stored at 4 °C.

### 2.4. Gene Polymorphism Analysis

A 3 µL aliquot of each PCR product was fractionated by agarose gel electrophoresis, visualized with gold view staining, and quantified using Tanon 3500 Gel Imaging (Tanon Biotech, Inc., Shanghai, China). The remaining PCR products were directly sequenced in both directions using a 3730*xl* DNA analyser (Applied Biosystems, Foster City, CA, USA) at Sangon Biotech Co. The sequencing results were analysed with DNAMAN 5.2 (Lynnon Corporation, San Ramon, CA, USA) and MEGA6.06 [14] to determine the SNPs of *IL-6* in Jinghai yellow chickens.

### 2.5. Bioinformatics Analysis of Interleukin-6 in Jinghai Yellow Chickens

#### 2.5.1. Prediction of the Promoter Region

The promoter is generally located in the 5′ upstream region of structural genes and can be divided into core, proximal, and distal promoter regions [15,16,17]. The promoter region is the basic transcription initiation complex or an important DNA element where RNA polymerase II binds and can precisely initiate and regulate messenger RNA transcription [18]. In this study, three types of bioinformatics software were used to predict the promoter region of IL-6: Promoter Scan [19], Neural Network Promoter Prediction (NNPP version 2.2) [20] and Promoter 2.0 Prediction Server [21].

#### 2.5.2. Transcription Factor Binding Site Prediction

Bioinformatics software was employed to predict possible transcription factor binding sites in the promoter region of chicken *IL-6* and corresponding transcription factors: AliBaba2.1 [22].

#### 2.5.3. Prediction of CpG Islands

A CpG island is a segment of DNA with high GC and CpG dinucleotide contents; CpG islands are usually located in the 5′ UTR (untranslated regions) of genes and are therefore often regarded as markers for the initiation of gene expression [23]. In this study, MethPrimer [24] bioinformatics software was used to predict CpG islands in the promoter region of chicken *IL-6*.

#### 2.5.4. Genetic Diversity Analysis

In this study, genotypic, allelic frequencies, and the Hardy Weinberg equilibrium for the newly discovered SNPs were calculated and tested using the formula by Wigginton et al. [25], and the polymorphism information content (*PIC*), heterozygosity (*H*), and effective number of allele (*Ne*) were calculated using the formula by Wigginton et al. [26].

## 3. Results

### 3.1. Single-Nucleotide Polymorphisms of Interleukin-6 in Jinghai Yellow Chickens

**Figure 1:**
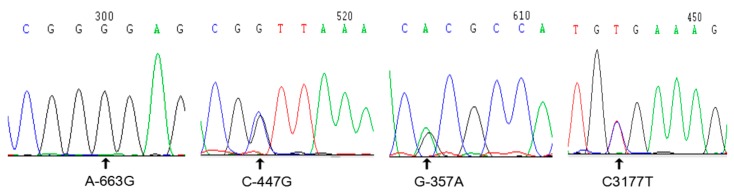
Sequencing results for newly discovered SNP sites.

A total of 28 SNP sites were detected (Table 2), among which 19 SNPs are located in the 5′ regulatory region, and three were newly discovered; the chromosomal positions of these three SNPs are 30948722, 30948938, and 30949028. Two of the SNPs are located in introns at the chromosome positions, 30951133 and 30951150. Two are located in exons at the chromosome positions, 30951621 and 30951826; both of these SNPs are synonymous mutations. Five SNPs are located in the 3′ region, one of which was newly discovered and is located at the position, 30952561. The sequencing results for the four newly discovered SNPs (Data S1), which are unlabelled in GenBank, are presented in Figure 1. Regarding the mutation at the −663 site, red jungle fowl exhibits an A at this position, whereas Jinghai yellow chickens exhibit a G. The other three SNPs are bimodally mutated heterozygous SNPs.

### 3.2. Population Genetic Variation of Newly Discovered Interleukin-6 Single-Nucleotide Polymorphisms 

The sequencing results indicate that only one GG genotype was detectable at the −663 (A > G) locus in Jinghai yellow chickens. As shown in Table 3, three genotypes, namely, CC, GG, and CG, were detected at the −447 (C > G) locus; GG, GA, and AA were detected at the −357 (G > A) locus in the promoter region of the *IL-6* gene; and CC, TT, and CT were detected at the 3177 (C > T) locus in the 3′ non-coding region. The χ^2^-test indicated that the newly discovered −663 (A > G) locus presents significant deviation from Hardy-Weinberg equilibrium, but the results for the −447 (C > G), −357 (G > A), and 3177 (C > T) loci were consistent with Hardy-Weinberg equilibrium (*p* > 0.05). The analysis of the polymorphic information indicated that the −447 (C > G), −357 (G > A), and 3177 (C > T) loci are moderately polymorphic (0.25 < *PIC* < 0.5). The chi-square independence test results showed that there was no significant difference in genotype frequency of the 28 SNP loci between different genders (*p* > 0.05).

### 3.3. Results of Prediction of the Interleukin-6 Promoter Region and Screening of Promoter Region Single-Nucleotide Polymorphisms 

In this study, three types of bioinformatic software were employed to predict the promoter region of chicken *IL-6*, and the results are presented in Table 4. Promoter Scan software predicted two promoter regions, with scores between 73.12 and 74.33. The Promoter 2.0 Prediction Server predicted one promoter region, which included only a starting site. Neural Network Promoter Prediction (NNPP version 2.2) predicted eight promoter regions, whose prediction scores were all above the 0.87, and the predicted score for two promoters was 0.99; therefore, the promoter predictions by the Neural Network Promoter Prediction (NNPP version 2.2) are more accurate than those of the two other software platforms. Taken together, the SNP mutation site information provided in Table 2 and the promoter prediction results shown in Table 4 demonstrate that the sites, 3, 4, 5, 11, 12, 13, 14, 15, 16, 17, and 19, all lie within the promoter region and that the loci, 11, 17, and 19, are newly discovered mutations. Therefore, these mutations might play a role in regulating the expression of *IL-6.*

### 3.4. Prediction of Putative Transcription Factor Binding in the Interleukin-6 Promoter by Single-Nucleotide Polymorphisms

The promoter region is a DNA sequence where RNA polymerase-specific recognition and binding occur. Nucleotide mutations in this region may change transcription factor binding sites, thereby affecting gene expression. The changes in transcription factors in the promoter region of *IL6* predicted by the online software AliBaba2.1 are presented in Table 5. According to the results shown in Table 5, a G > A mutation occurred at the newly discovered locus at −357 in the promoter region, resulting in an additional SP1 transcription factor binding site. The mutation at the −447 (C > G) locus, another newly discovered mutation, may lead to the disappearance of the original HB transcription factor binding site and the addition of a new C/EBPα transcription factor binding site. The mutation at the −458 (T > G) locus may lead to the disappearance of both the MEB-1 and GLO transcription factor binding sites and the addition of an SP1 binding site. The mutation at −483 (A > G) may lead to the disappearance of the original GATA-1 and C/EBPα transcription factor binding sites and the addition of ISGF-3, TEC1, and IRF-1 binding sites. At the −511 (C > T) site, the mutation may lead to the addition of an ICS BP transcription factor binding site, whereas the mutation at the −610 (G > A) site may lead to the disappearance of the original Sp1 binding site. The mutation at the −634 (A > G) locus may lead to the disappearance of the GRS and Sp1 transcription factor binding sites. At the −663 (A > G) locus, which was also newly discovered, the mutation may lead to the addition of binding sites for both the AP-2α and Sp1 transcription factors. At the −1280 (A > G) locus, the mutation may lead to another new binding site for the Sp1 transcription factor, whereas at the −1220 (G > T) locus, mutations may lead to the disappearance of the Sp1 and C/EBPβ transcription factor binding sites. At the −1223 (G > T) locus, mutation may lead to the disappearance of the C/EBPβ and C/EBPα transcription factor binding sites. Therefore, these mutations, especially at the newly discovered loci, may create new transcription factor binding sites and eliminate the original transcription factor binding sites, thereby affecting the regulation of *IL-6* expression.

### 3.5. Prediction Results for CpG Islands in the Promoter Region

**Figure 2:**
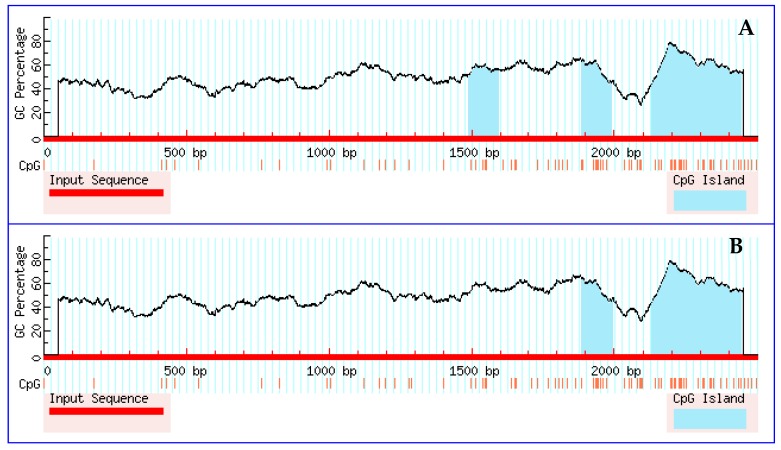
MethPrimer software prediction of changes in the CpG islands resulting from *IL-6* mutation. (**A**) The three CpG islands present before *IL-6* gene mutation, which are located at −1010–908 bp, −616–509 bp, and −370–58 bp. (**B**) The two CpG islands present after *IL-6* gene mutation, which are located at −615–505 bp and −370–58 bp.

To determine the effect of these SNPs on changes of the predicted CpG island in the promoter region of *IL-6*, MethPrimer software was used to predict the presence of CpG islands in the 2200-bp sequence upstream of the transcription start site of the *IL-6* promoter region before and after SNP site mutations. MethPrimer software (default parameter values for the software are CpG island length >100 bp, CG% > 50%, and Obs/Exp > 0.6) predicted three CpG islands before the occurrence of *IL-6* mutations located at −1010–908 bp, −616–509 bp, and −370–58 bp (Figure 2). However, two CpG islands were predicted after mutation of the *IL-6* gene, which are located at −615–(−505) bp and −370–(−58) bp, and only the −939 (C > G) mutation was detected in the CpG island region that disappeared. The comprehensive analysis of the transcription factor and CpG island prediction results showed that although SNP loci could lead to changes in the CpG island region, they did not cause transcription factor changes. This finding suggests that the −939 mutation site may affect gene expression through methylation, rather than through changes in promoter region transcription factors.

## 4. Discussion

Genetic diversity is an important component of the biodiversity of poultry, reflecting the degree of variation in the genetic information carried by organisms. DNA is the carrier of genetic information; therefore, changes in DNA directly reflect the degree of genetic variation among species. Genome-assisted breeding-based approaches for identifying molecular loci related to disease resistance are an important means of improving the resistance of poultry [27]. Genetic variation is vital for populations to adapt to varying environments and respond to artificial selection; therefore, any conservation and development scheme should start from an assessment of the state of variation in the population [28]. Across breeds, diversity is an important source of variation for rescuing problematic populations and new introgressed variants. An analysis of the genetic diversity of the three new loci found in the promoter region of *IL-6* demonstrated that the A−663G site is a homozygous mutation, where only the GG genotype was detected. Two alleles and three genotypes were detected at the −447 (C > G) site; the C gene allele frequency was 0.68, and the gene frequency of G was 0.32. Two alleles at the −357 (G > A) locus were detected, where the frequencies of G and A were 0.518 and 0.482, respectively. The information content of the two loci at −447 (C > G) and −357 (G > A) indicated moderate polymorphism. The chi-square adaptability test revealed that both the −447 (C > G) and −357 (G > A) loci were in Hardy-Weinberg equilibrium. This equilibrium may have been due to the Jinghai yellow chicken population reaching a stable genetic state after long-term breeding. However, the −663 (A > G) site significantly deviated from Hardy-Weinberg equilibrium, possibly because the number of selected samples was insufficient to fully reflect the phenotype of this locus in the whole population. Therefore, we must expand the sample size in the future to fully verify this result.

The 5′ regulatory region is important for the regulation of gene expression, and the identification of SNPs in this region is one approach for studying the associations between polymorphisms and characteristics, and for discovering new molecular markers [29,30]. Software programs may differ greatly in their predictions of the promoter region, transcription factor binding sites, and CpG island regions of the *IL-6* gene in chickens; therefore, to obtain more accurate results, we employed a variety of programs for this analysis to provide a basis for subsequent experimental verification. Promoters located directly upstream or at the 5′ end of transcription initiation sites are key regions in the regulation of eukaryotic transcription; the promoter plays a key role in gene expression and regulation, and is responsible for recruiting the transcription initiation complex to the point of origin of the gene, thereby initiating transcription [31,32]. Thus, base mutations in the DNA sequence of the *IL-6* gene promoter region may lead to abnormalities in the promoter transcription function. In this study, we found that 11 SNPs [−357 (G > A), 447 (C > G), 458 (T > G), 483 (A > G), 511 (C > T), 610 (G > A), 634 (A > G), 663 (A > G), 1208 (A > G), 1220 (G > T), and −1223 (G > A)] were located in the promoter region by predicting the promoter region of the *IL-6* gene, thus, providing the basis for the next step of verifying the effect of these SNP sites on transcription factor binding within the chicken *IL-6* gene.

Transcription factor binding sites are the DNA sequences to which transcription factors bind, and transcription is regulated by the interaction between these sites and transcription factors. Changes in binding sites can have significant effects on the binding of transcription factors to regulatory sequences and the expression products of genes [33]. Kogut et al. [34] reported that the Toll-like receptor agonists flagellin (FLG) and lipopolysaccharide (LPS) induce upregulation of *IL-6* and *CXCLi2* expression via the transcription factor, NF-κB, and AP-1-regulated extracellular signal-regulated kinases 1 and 2. In this study, we predicted the transcription factor binding sites of the *IL-6* gene using prediction software. All 11 SNP sites present in the promoter region of the *IL-6* gene were predicted to contribute to the addition, alteration, or disappearance of transcription factor binding sites. Among these SNPs, the newly discovered −357 (G > A) mutation site results in the addition of an Sp1 transcription factor binding locus at −360 bp. The −447 (C > G) mutation site leads to a transcription factor change (from HB to C/E BPα). The −663 (A > G) mutation site generates three new transcription factor binding sites (one AP-2α site and two Sp1 sites), whereas other SNP sites located in the promoter region lead to the generation of five new binding sites for transcription factors (Sp1, ISGF-3, TEC1, IRF-1, and ICS BP) and the disappearance of seven transcription factor binding sites (MEB-1, GLO, GATA-1, C/EBPα, Sp1, and GR sites and a second Sp1 site). In general, the length of a transcription factor binding site in the promoter region was 5 to 15 bp; therefore, it is necessary to analyse the transcription factor changes caused by the combinations of SNP sites located nearby. We analysed the regions and did not identify a combination of mutations that would lead to changes in transcription factor binding sites. The results of this study demonstrated that the Sp1 binding site appeared in multiple transcriptional binding regions after mutation. The binding of Sp1 to DNA activates transcription, and multiple copies of the Sp1 binding sequence are often distributed in the promoter or enhancer region of a gene. Sp1 regulates gene transcription by binding to DNA and interacting with other proteins [35,36]. Therefore, it is speculated that these SNP sites may affect the expression of *IL-6* genes through changes in Sp1 and other transcription factors.

CpG islands within a gene typically exist in the unmethylated state and can be bound by specific transcription factors to allow normal gene expression [37]. When methylation of CpG islands occurs, the transcription of the corresponding genes is silenced, thereby altering the expression of the genes [38,39]. DNA methylation can regulate gene expression by limiting the binding of transcription factors, and CpG islands are susceptible to methylation when the transcription factors of the CpG island region include Sp1, Myc, USF-1, CREB, CTCF, GATA-1, and AP-2, among others. After methylation, the binding efficiency at transcription factor binding sites is decreased, thereby reducing gene expression [40,41]. In this study, CpG islands in the promoter region of the chicken *IL-6* gene were predicted with software, and the CpG islands differed before and after *IL-6* gene mutation. In the −1010-908 bp region, due to the presence of the −939 mutation site, one less CpG island was present after mutation. This prediction suggests that the degree of methylation in the promoter region of the *IL-6* gene is likely to affect the expression of the gene, however, this conclusion requires further validation in subsequent studies.

## 5. Conclusions

In this study, the *IL-6* gene sequences of 220 Jinghai yellow chickens were analysed. Twenty-eight SNPs were identified in the region −2200 bp upstream to 500 bp downstream of the chicken *IL-6* gene. A comparison of these SNPs with the NCBI database (https://www.ncbi.nlm.nih.gov/) identified three new SNP sites [−357 (G > A), 447 (C > G), and −663 (A > G)] in the promoter region of the 5′ regulatory region of the gene, while one new SNP site [3177 (C > T)] was identified at 482 bp in the 3′ non-coding region of the gene, and these four SNPs are not labelled in GenBank. Among these sites, both −447 (C > G) and −357 (G >A) exhibit Hardy-Weinberg equilibrium and are rich in polymorphic information, whereas −663 (A > G) significantly deviates from Hardy-Weinberg equilibrium. The remaining novel SNP is located in the 3′ non-coding region. There are 11 SNPs located in the promoter region of the 5′ regulatory region, and these SNPs might lead to changes in transcription factor binding sites and CpG islands, thereby altering gene expression. These predictions provide a basis for further study of the regulation of *IL-6* expression and its relationship to chicken coccidiosis.

## Figures and Tables

**Table 1:** Primer sequences used for PCR amplification of chicken interleukin-6 (IL-6).

Primer	Starting Position	Primer Sequence (5′ → 3′)	Annealing Temperature (°C)	Length (bp)
P1	−2210	F: AGAGAGGACTAACCCACAGAG	58.5	698
		R: CCAGCTTCTCCAGTCTTGTC		
P2	−1628	F: AGGGACAGCAATGGCAGAAG	60.5	717
		R: AAGAGCTGATCCTGGTTCTGG		
P3	−994	F: CAGAGGACGTCCTACCTCAA	56.5	690
		R: GGTGAGCCTGGCAGCC		
P4	−367	F: AAGATAAGACGCGCCACACC	58.5	803
		R: TTGAGGTTGTTCCGGACGAG		
P5	391	F: CGTGTGCGAGAACAGCAT	59.0	565
		R: AAATAGAAAGTTGGAAGGAGAGTACA		
P6	797	F: GGTGTGCAGTCGGCAATAG	57.5	803
		R: CTCATCATTCCACACAAGG		
P7	1388	F: ACTGTGTGCTCCTAATGCCT	60.5	770
		R: CTTCAGATTGGCGAGGAGGG		
P8	1989	F: CGTACCTCAAAACCTACCTCAG	59.0	768
		R: AGTGGAGTTCTTCAGCCTTATTTAA		
P9	2702	F: CAAAACCAACCTGTCTAAGCTG	59.5	773
		R: TTTTAAGAACTGTATGTTGATTGTGC		

Bp: base pairs, F: Forward, R: Reverse.

**Table 2:** Information for *IL-6* single-nucleotide mutation sites.

Mutation Site ^1^	Chromosome Position	Serial Number	SNP Locus
1	30947851	rs315131240	−1534 (T > C)
2	30948105	rs731482590	−1280 (C > T)
3	30948162	rs15937151	−1223 (G > A)
4	30948165	rs15937152	−1220 (G > T)
5	30948177	rs15937153	−1208 (A > G)
6	30948446	rs316873841	−939 (C > G)
7	30948496	rs316713313	−889 (G > A)
8	30948520	rs15937155	−865 (G > A)
9	30948594	rs732353671	−791 (G > C)
10	30948651	rs732784937	−734 (G > A)
11	30948722		−663 (A > G)
12	30948751	rs317547243	−634 (A > G)
13	30948775	rs317511948	−610 (G > A)
14	30948874	rs740913355	−511 (C > T)
15	30948902	rs314710056	−483 (A > G)
16	30948927	rs794072580	−458 (T > G)
17	30948938		−447 (C > G)
18	30948985	rs317677898	−400 (A > G)
19	30949028		−357 (G > A)
20	30951133	rs737499962	1749 (C > T)
21	30951150	rs736574918	1766 (G > A)
22	30951621	rs315658915	2237 (A > G)
23	30951826	rs794520257	2442 (T > C)
24	30952410	rs317567286	3026 (G > A)
25	30952457	rs794365857	3073 (C > T)
26	30952561		3177 (C > T)
27	30952578	rs316333207	3194 (C > T)
28	30952600	rs740039941	3216 (C > G)

^1^ The mutation sites, 1~19, are located in the 5′ regulatory region; the mutation sites, 20 and 21, are located in the third intron; the mutation sites, 22 and 23, are located in the fourth exon; and the mutation sites, 24~28, are located in the 3′ regulatory region. SNP; single-nucleotide polymorphism.

**Table 3:** Population genetic analysis of the newly discovered *IL-6* SNPs −663 (A > G), −447 (C > G), −357 (G > A), and 3177 (C > T).

No	Mutation Site	Sample Size	Genotype Frequency ^1^			Gene Frequency		χ^2^	*p*-Value	*PIC*	*H*	*Ne*
1	A-663G	220	AA: 0.000 (0)	GG: 1.000 (220)	AG: 0.000 (0)	A: 0.000	G: 1.000			0.000	0.000	1.000
2	C-447G	220	CC: 0.455 (100)	GG: 0.090 (20)	GC: 0.455 (100)	C: 0.680	G: 0.320	0.500	0.799	0.340	0.434	1.766
3	G-357A	220	GG: 0.290 (64)	AA: 0.255 (56)	GA: 0.455 (100)	G: 0.518	A: 0.482	1.770	0.413	0.375	0.499	1.997
4	C3177T	220	CC: 0.400 (88)	TT: 0.145 (32)	CT: 0.455 (100)	C: 0.627	T: 0.373	0.170	0.918	0.358	0.468	1.878

^1^ The number in brackets is the sample size. *PIC*: Polymorphism information content, *H*: Heterozygosity, *Ne*: Effective number of allele.

**Table 4:** Prediction scores of the *IL-6* gene promoter region.

Prediction Software ^1^	Start Site (bp)	Termination Site (bp)	Score
Promoter Scan [19]	−402	−652	73.12
	−74	−324	74.33
Promoter 2.0 Prediction Server [21]	−900	-	1.092
Neural Network Promoter Prediction (NNPP version 2.2) [20]	−330	−380	0.87
	−339	−389	0.97
	−519	−569	0.99
	−659	−709	0.99
	−1049	−1099	0.92
	−1192	−1242	0.97
	−1404	−1454	0.91
	−2179	−2229	0.87

^1^ The scores range from 0 to 100 for Promoter Scan software, and higher scores reflect a higher prediction accuracy. A score below 0.5 obtained using Promoter 2.0 Prediction Server software represents an inexact prediction; 0.5–0.8, a marginal prediction; 0.8–1.0, a moderately likely prediction; and a score above 1.0, a highly likely prediction. The range of scores is 0 to 1 for the Neural Network Promoter Prediction (NNPP version 2.2), and a higher score reflects a higher prediction accuracy.

**Table 5:** Changes in transcription factors before and after SNP mutation in the promoter region of the chicken *IL-6* gene.

Mutation Site	Base Group	Transcription Factor	Transcription Factor Binding Site Base Sequence	Transcription Factor Position
−357	G	Sp1	gacacgccacacc	−360–(−348)
	A			
−447	C	HB	cttaaaaacg	−447–(−438)
	G	C/EBPα	aaacggttaa	−452–(−443)
−458	T	MEB-1 GLO	ttttttaaac ttttttaaac	−458(−449) −458–(−449)
	G	Sp1	ccgaggaggg	−467–(−458)
−483	A	PU.1 GATA-1 C/EBPα	aaaagagaac aaatgaaaag acaaatgaaa	−483–(−474) −488–(−479) −490–(−481)
	G	PU.1 ISGF-3 TEC1 IRF-1	gaaagagaac ggaaagagaa ggaaagagaa ggaaagagaa	−483–(−474) −484–(−475) −484–(−475) −484–(−475)
−511	C	TBP	tgcttataag	−513–(−504)
	T	TBP ICS BP	atgtttataa agtttcatgt	−514–(−505) −520–(−511)
−610	G	Sp1	ggagcgatgg	−615–(−606)
	A			
−634	A	GR Sp1	cactgtgtgc cctccaccact	−635–(−626) −642–(−632)
	G			
−663	A			
	G	AP-2α Sp1 Sp1	cccggggagc gccccggggag cctgctgccccggg	−668–(−659) −670–(−660) −676–(−663)
−1208	A	Sp1	ggctcggggagccag	−1221–(−1207)
	G	Sp1	ggggagccgggc	−1216–(−1205)
−1220	G	Sp1 C/EBPβ	ggctcggggagccag gctgtggctc	−1221–(−1207) −1226–(−1217)
	T			
−1223	G	C/EBPβ C/EBPα	gctgtggctc ataattgctg	−1226–(−1217) −1232–(−1223)
	A			

## Supplementary Materials

The following are available online at http://www.mdpi.com/2073-4425/9/9/446/s1. Data S1: Sequencing results for newly discovered SNP sites; File S1: Sequence of the newly discovered SNPs in *IL-6* gene before and after mutation.
