# Supplementary material for: Bioinformatics Analysis of SNPs in IL-6 Gene Promoter of Jinghai Yellow Chickens
Source: Genes (Basel). 2018 Sep 6;9(9):446. doi: 10.3390/genes9090446 (PMC6162446; doi:10.3390/genes9090446)
Supplement: Supplementary file 1 [file genes-09-00446-s001.zip › Supplementary materials/Supplementary File S1.docx]

**NCBI original sequence of gallus**

GTCTGACAGAGGACGTCCTACCTCAACTCTTGTGCCAACGTCGCCCTGCGCCCCGTGCCC

TCCCCATTCCAGAACCAGGATCAGCTCTTTCTCTGTTCTTCTCTATTAGGCGAAATCACA

GGGTGACACTCCAAAGGCCGACCAGCAGCACGCCCCGGGCAGCTCCCCAAAGCCCATGTC

AGCCTGTACCTGAGCTGAATGGGCACACTGGTTCTGGGCAAAGAATCCCTCAGCGAAAGG

CCAAAATTCCCAGCACCCCTCCCTGTGTGGCGAAGGAGATAAGGCTGCAGATAGCGGGTA

AATAACTGCGTGGCATTTCCCGTGCCTGCTGCCCCGG**A**GAGCCCTGGGTGCTGACCTTCC

TCCACCACTGTGTGCTGGAAACACCGGAGCGATGGGATGTGCCCAGGTGTGGTTCACAGA

GGGGTGCGCGTGTGACGGCGTATAACGTGGACGTGTGCAGCGGCTGAACCAAGGGCGTCC

AGTTTCATGCTTATAAGTAATAAAAACAGAACAAATGAAAAGAGAACACCAGCCCGAGGA

GGTTTTTTAAACG**C**TTAAAAACGAGAATAAAGATGCATAACGAATAAAAGTCGCGAAAAC

ATATCAATGGATAAGATGTATAAAACACAATAAAAGATAAGAC**G**CGCCACACCTGTGCGC

AGGTACGGGACCCTGCAGGAGGCTGCCAGGCTCACCCCCCGCCCCGACCCCCGGCCGAAG

CCAGGTGAGCGGCGGCGCGGCCCGCAAGGAGTTACGGGGAGGAAAAATGACTTCATGCCT

CTGCCTGGCCTGGCTCGCCTCCCCCTCCCCGTCTCCGTTTCACAATCTCAATGCTCTCGG

TTCATCAACACCTGCTGAATGTTTGTGGAGACCAAACAGTGGATGTGAGCACAGTGAGGC

AGTGGGTGATGTGTTTCAGCAATGGCAACAGCAATG**C**GAAAGACAAACCATGGCGTGGAT

GGCCATGCACAGCTGCCATGCTACAAAATGAAGAGCATCTTGATCAACTTATCCACCTGT

ATTGGCTAATAATGGTGGTGACTATGTTGAAATAC

Jinghai Yellow Chickens Sequence

GTCTGACAGAGGACGTCCTACCTCAACTCTTGTGCCAACGTCGCCCTGCGCCCCGTGCCC

TGCCCATTCCAGAACCAGGATCAGCTCTTTCTCTGTTCTTCTCTATTAGGCAAAATCACA

GGGTGACACTCCAAAAGCCGACCAGCAGCACGCCCCGGGCAGCTCCCCAAAGCCCATGTC

AGCCTGTACCTGAGCTGAATGGGCACACTCGTTCTGGGCAAAGAATCCCTCAGCGAAAGG

CCAAAATTCCCAGCACCCCTCCCTGTATGGCGAAGGAGATAAGGCTGCAGATAGCGGGTA

AATAACTGCGTGGCATTTCCCGTGCCTGCTGCCCCGG**G**GAGCCCTGGGTGCTGACCTTCC

TCCACCGCTGTGTGCTGGAAACACCGGAGCAATGGGATGTGCCCAGGTGTGGTTCACAGA

GGGGTGCGCGTGTGACGGCGTATAACGTGGACGTGTGCAGCGGCTGAACCAAGGGCGTCC

AGTTTCATGTTTATAAGTAATAAAAACAGAACAAATGGAAAGAGAACACCAGCCCGAGGA

GGGTTTTTAAACG**G**TTAAAAACGAGAATAAAGATGCATAACGAATAAAAGTCGCGAAAAC

GTATCAATGGATAAGATGTATAAAACACAATAAAAGATAAGAC**A**CGCCACACCTGTGCGC

AGGTACGGGACCCTGCAGGAGGCTGCCAGGCTCACCCCCCGCCCCGACCCCCGGCCGAAG

CCAGGTGAGCGGCGGCGCGGCCCGCAAGGAGTTACGGGGAGGAAAAATGACTTCATGCCT

CTGCCTGGCCTGGCTCGCCTCCCCCTCCCCGTCTCCGTTTCACAATCTCAATGCTCTCGG

TTCATCAACACCTGCTGAATGTTTGTGGAGACCAAACAGTGGATGTGAGCACAGTGAGGC

AGTGGGTGATGTGTTTCAGCAATGGCAACAGCAATG**T**GAAAGACAAACCATGGTGTGGAT

GGCCATGCACAGCTGGCATGCTACAAAATGAAGAGCATCTTGATCAACTTATCCACCTGT

ATTGGCTAATAATGGTGGTGACTATGTTGAAATAC
